# Dose–response Relationship of Reported Lifetime Meditation Practice with Mental Health and Wellbeing: a Cross-sectional Study

**DOI:** 10.1007/s12671-022-01977-6

**Published:** 2022-09-28

**Authors:** Nicholas I. Bowles, Jonathan N. Davies, Nicholas T. Van Dam

**Affiliations:** Contemplative Studies Centre, Melbourne School of Psychological Sciences, The University of Melbourne, Melbourne, Australia

**Keywords:** Dose, Response, Meditation, Mindfulness, Vipassana

## Abstract

**Objectives:**

Meta-analyses of meditation studies have revealed mixed modest evidence of benefits across a range of outcomes. However, because this evidence-base is predominantly from brief interventions, it is unclear whether it accurately reflects how contemporary meditators practice or the dose–response relationship between amount of practice and outcome. This study sought to characterize how contemporary meditators practice, examine any possible dose–response relationships between historical practice and measures of psychological wellbeing, and explore which characteristics of practice most strongly predict favorable psychological outcomes.

**Methods:**

One thousand six hundred and sixty-eight meditators (*M* = 1095 h practice, *SD* = 2365) responded to advertisements in meditation practice communities and social media. We explored associations between demographics, meditation practice characteristics, and outcomes including positive and negative affect, psychological distress, and life satisfaction in a cross-sectional study design.

**Results:**

Historical meditation practice (accumulated lifetime hours) was significantly associated with favorable psychological outcomes (|*r*| ranging from .18 to .28). Model fit was optimized with a generalized additive model (average increase in *R*^2^ = 2.22), indicating non-linear effects. The strength of association between practice time and outcomes was generally strongest for approximately the first 500 h, before plateauing. Several practice types including Vipassana (as taught by S.N. Goenka) and cultivating practices (e.g. compassion, lovingkindness) were more strongly predictive of favorable psychological outcomes.

**Conclusions:**

Benefits of meditation accrue over time in a non-linear manner, and show variation based on practice context. These results highlight the importance of understanding how the benefits of meditation accrue over longer time durations than typical standardized programs.

**Supplementary Information:**

The online version contains supplementary material available at 10.1007/s12671-022-01977-6.

Meditation is an umbrella term that describes many contemplative practices across a variety of contexts, used for a variety of different goals. Within the Buddhist context for example, meditation is generally considered a (multiple) lifetime pursuit toward awakening (Anālayo, 2004). Within this context, meditation has a long-term orientation and is often delivered under the supervision of an experienced teacher in regular and/or intensive retreat settings (Lutz et al., 2004). Within Buddhism, popularization of meditation for the laity (i.e. non-monastic practitioners) is a relatively modern phenomenon, promoted in the late nineteenth and early twentieth centuries (e.g. Braun, 2013). Even so, it was generally assumed that one would be practicing within a religious or spiritual context.

Since then, meditation has gained steady mainstream appeal, with various modifications to its form and goals (e.g. McMahan, 2008) increasing accessibility among those outside of religious and spiritual settings. Recent decades have seen especially pronounced growth, more than threefold in US adults from 2012 (4.1%) to 2017 (14.2%) (Clarke et al., 2018). This mainstreaming has come largely off the back of the first wave of Western “enlightenment seekers”, who returned from Asia in the 1970s to teach a secularized version of meditation for spiritual growth (Goldstein & Kornfield, 2001). Over time however, these meditation techniques increasingly appealed more for their therapeutic value than as a path to enlightenment. Packaged now as therapeutic modalities, mindfulness-based stress reduction and other secular mindfulness-based programs (MBPs, Crane et al., 2017) assumed a far shorter-term orientation and were delivered in group settings with relatively lower intensity compared to traditional practices (Kabat-Zinn, 2011). A further reduction of timescale has occurred alongside the recent proliferation of online mindfulness-based programs, including mindfulness apps. Amidst a crowded market of commercial offerings, many apps have promised comparable gains for lesser time investments (Marshall et al., 2020).

One by-product of the creation of MBPs — and subsequently, meditation apps — has been the implicit assumption that there is an identified dose–response relationship between practice “dose” and the accumulation of benefits on a range of outcomes and processes. Early case–control studies comparing expert meditators against non-meditators on outcomes including pain, attention, cognition, and ageing (Lykins & Baer, 2009) seemed to support the benefits of lengthy practice (selection concerns notwithstanding; e.g. Davidson and Kaszniak, 2015). However, the evidence for dose–response relationships among shorter interventions based on outcomes from randomized controlled trials of MBPs has been far less convincing. Such evidence within the context of meditation apps is essentially non-existent.

Empirical evidence suggests meditation has modest benefits for common mental health conditions like anxiety and depression, in both clinical (Baer, 2003; Goyal et al., 2014; Strauss et al., 2014; Teasdale et al., 2000) and non-clinical populations (Galante et al., 2021; Khoury et al., 2015), as well as some domains of cognitive function (Lao et al., 2016; Whitfield et al., 2022). These benefits are observed most robustly in MBPs, which have been widely used and researched (Dimidjian & Segal, 2015). A recent meta-analysis of MBPs (*k* = 203) reported overall post-program effect sizes against inactive controls for psychological conditions of *d* =  − 0.49 for anxiety, *d* =  − 0.73 for depression, and *d* =  − 0.73 for stress (Strohmaier, 2020). Against active controls, effect sizes reduced to *d* =  − 0.16 for anxiety, *d* =  − 0.20 for depression, and *d* =  − 0.33 for stress (Strohmaier, 2020). Such effects relate to the overall program content, which includes several curriculum elements (training and practice of mindfulness meditation, teacher-led instruction sessions, group discussions, daylong retreat), all of which likely contributing to the programs’ beneficial outcomes (Canby et al., 2021).

The relative standardization of MBPs (typically 6–10 weeks, with 40 to 45 min of daily meditation, see Strohmaier, 2020) and presence of multiple active ingredients have posed challenges for accumulating evidence for dose–response effects in this context. A meta-analysis by Parsons et al., (2017) of mindfulness-based stress reduction (MBSR) and mindfulness-based cognitive therapy (MBCT) found a small but significant association between *self-reported home practice* amount and favorable psychological outcomes for conditions such as anxiety, depression, and stress (*r* = 0.26, 95% confidence interval (C.I.) 0.19 to 0.34). By contrast, a recent meta-analysis from Strohmaier (2020), which included MBSR, MBCT, and other MBPs, found no statistically significant dose–response relationship between *recommended home practice* and the same psychological conditions.

As MBPs move to more asynchronous, online-based formats (Jayawardene et al., 2017), they are being offered with shorter and shorter durations at the expense of many beneficial elements such as teacher-led sessions, group discussions, and retreats (Spijkerman et al., 2016). Two meta-analyses of online MBPs reported reduced effect sizes against mostly inactive controls, compared to those delivered in traditional standardized formats, for perceived stress (*g* =  − 0.43 to *g* =  − 0.51) (Jayawardene et al., 2017; Spijkerman et al., 2016), anxiety (*g* =  − 0.22), depression (*g* =  − 0.29), and wellbeing (*g* = 0.23) (Spijkerman et al., 2016). These meta-analyses suggest that as MBPs become shorter, asynchronous, and less interactive, they compromise on efficacy relative to traditional (spiritual) meditation practice or standardized MBPs (Canby et al., 2021).

However, despite the apparent drawbacks of online programs, they offer a number of advantages in terms of high accessibility (Spijkerman et al., 2016), which are particularly leveraged in the context of mobile apps (Gál et al., 2021). App-based meditation programs typically feature even lower doses of meditation than MBPs, with introductory programs including approximately 10 min of daily practice time for between 10 and 30 days (Gál et al., 2021). A meta-analysis of app-based meditation programs (*k* = 34) reported effect sizes against inactive controls of *g* =  − 0.31 for anxiety, *g* =  − 0.35 for depression, *g* =  − 0.62 for stress, and *g* = 0.31 for wellbeing, while effect sizes against active controls were not significant (Gál et al., 2021). These findings, like online MBPs, are generally of a smaller magnitude to those reported for in-person MBPs.

The increasing predominance of both standardized MBPs and more recently online programs raises questions about how representative existing empirical research is of the way most people practice meditation. Empirical research of app-based programs to date has included only 15 different apps (Gál et al., 2021), which represents a small fraction of the hundreds of such programs available (Mani et al., 2015). And while it is unknown exactly what practices are featured across available app-based programs, the range of practices in MBPs is relatively narrow when compared to the multitude of meditation practices available across active practice traditions (Matko & Sedlmeier, 2019). And while MBPs generally entail a time commitment of less than 100 h (Strohmaier, 2020), their original intention was primarily to foster a sustainable and autonomous lifelong practice (Kabat-Zinn, 1982, 2011). The effectiveness of MBPs, however, is almost universally based on measures collected at program completion or, in a small number of cases, a 3- to 6-month follow-up period. It is yet unknown whether such programs lead to sustainable ongoing practice, and whether (and to what degree) benefits continue to accrue during phases of self-directed practice over longer time durations.

One way to provide a broader perspective on dose-response relationships, and the longer-term benefits of short, online, or app-based programs, is to conduct a broadly representative cross-sectional study of contemporary meditators with differing levels of practice experience across a range of practice modalities, motivations, and orientations. This approach would overcome biases inherent to meta-analysing empirical data, and the associated differences in content and delivery features that emerge as practices become shorter. Furthermore, it allows estimates of the dose-response relationship overall and for specific modalities, such as traditional Buddhist practice, traditional group-based MBP, online MBP, and app-based programs. In an analogous cross-sectional model in the field of psychotherapy, there is broad support for a log-linear dose-response relationship between outcomes and time whereby the benefits of treatment are greatest in the early stages before plateauing over longer durations (Robinson et al., 2020).

Therefore, in the present study, we sought to develop a dose-response model for meditation practice over a longer time duration than MBPs and other frequently employed short-term interventions. The three aims of the study were, first, to examine the demographic, clinical, and practice characteristics of a broad sample of contemporary meditation practitioners; second, to explore the relationship between historical practice and psychological outcomes over longer durations than typical short-term interventions; and third, to explore which practice characteristics best predict psychological outcomes and thus mediate a dose-response relationship.

## Method

### Participants

One thousand six hundred and sixty-eight adults (aged 18–75) completed the first section of the survey (Block 1, see “Measures”) and were included in the study. To be included, participants had to have an existing regular meditation practice or an intention to establish a regular meditation practice and be fluent in English. We excluded 236 respondents who commenced the survey: 164 provided inadequate data (i.e. no information on practice history, no indication of whether the participant meditated in the past month or if they did, the frequency or duration); 65 reported mutually incompatible responses (i.e. no history of active practice *and* practice types they use and/or their prior participation in a multi-day retreat); 7 indicated they engaged in only movement or breathing contemplative practices such as walking meditation, yoga, pranayama, and Tai Chi/Qi Gong. A further 115 participants did not complete Block 2 (see “Measures”) and were excluded from the main analyses.

Demographic and practice characteristics for the 1668 participants are reported in Table 1. The mean age of participants was just over 45 years (*SD* = 15.2) and nearly three-quarters (69.9%, *n* = 1166) of participants were female. Just over half of participants (50.8%, *n* = 848) resided in Australia and just over a quarter in the USA (27.3%, *n* = 456). More than a quarter of participants (27.3%, *n* = 456) reported a diagnosed mental health or neurological condition, with 45.9% of those participants reporting an anxiety-related condition, 43.1% a depression-related condition, and 24.0% reporting a condition related to both anxiety and depression. Most participants (91.4%) reported having an active meditation practice while 5.7% reported having some prior meditation experience but no recent (within the last month) practice. The average duration of active practice was 5.8 years (median = 3, *SD* = 7.7) while participants, on average, had 1095.5 h (median = 266.0, *SD* = 2364.8) of accumulated lifetime practice. For the analysis that follows, upper-end outliers for these two measures of historical practice (representing *n* = 82 participants) were adjusted to the 95th percentile values of 20 years of active practice and 4715 accumulated hours. Just over half of participants (54.2%) reported using a mobile application to support their practice, while 40.2% reported prior participation in a multi-day silent retreat. And for self-assessed practice need, participants mostly believed they needed to practice daily or near daily to achieve the goals of their practice (*M* = 6.1, *SD* = 1.3) and while there was significant variation in the time per day participants believe they needed, based on experience and the relative strength of different practice goals, participants overall believed they needed just over half an hour of practice per day (*M* = 37.6, *SD* = 40.2).

**Table 1:** Demographic and practice characteristics of 1668 participants

Measure	*n*	Proportion	Mean (*SD*)
Age			45.4 (15.2)
Gender — female	1166	69.9%	
Diagnosed mental health or neurological condition	455	27.1%	
Resides in Australia	848	50.8%	
Resides in USA	454	27.3%	
Resides in UK	140	8.4%	
Duration of active practice (years)			5.8 (7.7)
Accumulated lifetime practice time (hours)			1095 (2365)
Prior meditation experience — active practice	1524	91.4%	
Prior meditation experience — inactive practice	95	5.7%	
Prior meditation experience — none	49	2.9%	
Multi-day retreat experience — yes	670	40.2%	
Use of meditation mobile app	904	54.3%	
Self-perceived practice need — days per week			6.1 (1.3)
Self-perceived practice need — time per day (mins)			37.6 (40.2)
Overall duration — lifetime		83.1%	

To determine whether we were successfully recruiting a representative sample of contemporary meditation practitioners, we conducted a 1-month wave of targeted Facebook advertising, which represents 15% of our total sample. Chi-squared tests revealed were no significant differences in most key demographic and practice characteristics (see Supplementary Table S1) with two exceptions — age and retreat experience, with the Facebook-recruited group being older (*χ*^2^(57) = 77.8, *p* = 0.035) but less likely to have retreat experience (*χ*^2^(1) = 27.71, *p* =  < 0.001). These results suggest our convenience sample was targeting appropriately, although given that our recruiting efforts included meditation centres, practitioners with retreat experience may be over-represented in our sample.

### Procedure

Data were collected via an anonymous online survey, which consisted of three blocks (described below). The survey was open between April 2020 and September 2021 and hosted on the Qualtrics online survey platform. The survey link was posted to various online public forums (e.g. Reddit) and social media channels (e.g. Facebook, Twitter) along with a short introduction to the project. Direct email contact was also made with representatives of various meditation practice communities and centres, along with a request to promote the study among their members. Recruitment was supplemented by a Facebook advertising campaign, with an interest-based targeting strategy using the keywords: “meditation” and “mindfulness”. Recruitment focused on Australia, the USA, and the UK, although participants residing in other countries were also able to participate.

Participants provided informed consent to participate and then completed the survey, which took approximately 5 min for each of the three blocks (i.e. total time approximately 15 min). All participants (*n* = 1668) completed Block 1 (demographics, mental/neurological health, meditation practice history, life satisfaction, stressful events). Participants were then given the option of completing Block 2 (*n* = 1553; psychological distress, positive affect, and negative affect) and Block 3 (*n* = 1393; personality traits). By completing Block 1, participants were entered into a draw for 1 of 10 gift cards to the value of AU$100.

### Measures

The five primary outcomes (assessed in Block 2) were psychological distress, positive affect, negative affect, affect balance, and satisfaction with life. However, we present the measures used in block order for the sake of clarity.

#### Demographics and Meditation Practice History (Block 1)

*Demographic data* included age, gender, country of residence, and presence/absence of a diagnosed mental health or neurological condition.

*Meditation practice history* questions included (1) duration of active practice (years and months); (2) contemplative practices (selected from a list) regularly used; (3) the faith/tradition (selected from a list) the participant practices within; (4) the relative strength of different practice goals on a scale of importance ranging from 1 = “low importance” to 100 = “extremely important” (50 = “moderately important”); (5) the frequency and duration of practice the participant believed they need to achieve their goals (self-assessed practice need); (6) prior participation in an multi-day silent meditation retreat; (7) whether the participant uses a mobile application to help practice and if so, which one (selected from a list); (8) whether the participant practiced meditation in the past month and if so, (8.1) the average frequency and duration of those sessions; (8.2) how that amount compares to prior periods of active practice; (8.3) the proportion of guided versus unguided sessions in the past month; (8.4) the tools and methods used to support practice in the past month (e.g. books, mobile app); and (8.5) whether the participant meditated at a regular time each day.

Accumulated lifetime practice hours were calculated as:

1 $$(\left(d*t\right)*\left(4.345\right)*p*\left(n-1\right))+(\left(d*t\right)*4.345))+(r*8)$$

where *d* = reported practice days per week in prior month, *t* = reported average session duration per week in prior month, 4.345 = the average number of weeks per month, *p* = a multiplying factor based on how reported practice in prior month compares to past periods of active practice, *n* = reported months of lifetime active practice, and *r* = total days spent on a multi-day silent retreat (8 h of formal practice was counted per day of retreat in line with Lutz et al., 2004). Participants were also asked whether they had experienced a major stressful life event in the past month and if so, to report whether that event was related to COVID-19, and to provide a subjective rating (on a scale of 1 to 5, where 1 = “a little stressful” and 5 = “extremely stressful”) of how stressful they found the event. The full questionnaire and calculations are available in Supplementary Material (osf.io/zbqdh/).

#### Psychological Distress and Affect (Block 2)

*Kessler Psychological Distress Scale* (K10; co-primary outcome (Kessler et al., 2002). Psychological distress was measured using the K10, a 10-item questionnaire assessing psychological distress with questions about anxiety and depressive symptoms experienced over the prior 4-week period. Each item has a 5-point Likert scale ranging from 1 = “none of the time” to 10 = “all of the time”. It has excellent (0.93) internal consistency in similar research (Kessler et al., 2003). Internal reliability coefficients in the present sample were high (Cronbach’s *α* = 0.9, McDonald’s *ω* = 0.93).

*Satisfaction with Life Scale, Single Item* (SWLS, co-primary outcome (Cheung & Lucas, 2014; Diener et al., 1985). Life satisfaction was measured with a single question: “Over the past month, how satisfied have you been with your life?” The question has a 4-point Likert scale ranging from 1 = “very satisfied” to 4 = “very dissatisfied”. Items were reverse coded so that high scores indicated higher levels of life satisfaction.

*Scale of Positive and Negative Experience (SPANE)* (Diener et al., 2009). Positive affect, negative affect and affective balance (co-primary outcomes) were measured using SPANE, a 12-item questionnaire assessing the frequency of positive and negative affect and related affective balance experienced in the past 4 weeks. Each item has a 5-point Likert scale ranging from 1 = “very rarely or never” to 5 “very often or always”. It has excellent ($\alpha$ = 0.81–0.89) internal consistency in similar research (Diener et al., 2009). Internal reliability coefficients in the present sample were high (Cronbach’s *α* = 0.92, McDonald’s *ω* = 0.94).

#### Personality Traits (Block 3)

*Big Five Inventory-2 Short Form (BFI-2-S)* (Soto & John, 2017a). Personality traits were measured using the BFI-2-S, a 30-item questionnaire measuring personality at the domain and trait level. Five domains are assessed: open-mindedness, conscientiousness, extraversion, agreeableness, and negative emotionality, each of which contains three facets. It has excellent ($\alpha$=0.79–0.89) internal consistency in similar research (Stewart et al., 2021). In the present sample, internal reliability was acceptable for each of the domains (open-mindedness: Cronbach’s *α* = 0.74, McDonald’s *ω* = 0.85, conscientiousness: Cronbach’s *α* = 0.78, McDonald’s *ω* = 0.85, extraversion: Cronbach’s *α* = 0.74, McDonald’s *ω* = 0.85, agreeableness: Cronbach’s *α* = 0.73, McDonald’s *ω* = 0.84, negative emotionality: Cronbach’s *α* = 0.86, McDonald’s *ω* = 0.92).

### Data Analyses

Data cleaning was first conducted to exclude participants who failed to provide basic demographic information (i.e. age, gender, country of residence). Duplicate responses were removed, with the more complete or (if same) more recent response being retained. Participants were excluded from the analysis if they (1) provided inadequate information on their practice history and recent practice (i.e. whether they meditated in the past month and if so, frequency and duration) or the type of practice they used; (2) reported mutually incompatible responses to different questions (e.g. no meditation experience but prior participation in a meditation retreat); and (3) reported that they only used movement or breathing practices.

Psychological outcome variables (i.e. affect and psychological distress) were scored as per published guidelines and analysed at the total score level. All dependent variables approximated a normal distribution (skew <|0.50|) except for psychological distress, which was positively skewed (skew = 1.01). A separate analysis was conducted on the log-transformed psychological distress data, which was approximately normally distributed (skew = 0.29). Participants with any item missing for psychological distress or affect were excluded from subsequent analyses for that relevant variable only. Average scores for each of the personality domains were calculated, and participants with more than one item missing on any domain were excluded from subsequent analyses for that relevant domain only.

As a key predictor of dose–response relationships, dependent variables measuring historical practice (i.e. duration of active practice and accumulated lifetime practice hours) were checked for the presence of outliers at the upper end (lower end values commenced at zero for novice practitioners). Outliers for both years/months and hours were transformed with the Winsorization method, using the Winsorize function in the DescTools package in R (Signorell, 2021).

To explore the association between practice characteristics and outcome measures, we first assessed correlations between different measures of historical practice and outcome measures. Historical practice was expressed as (i) duration of active practice; (ii) accumulated lifetime practice hours; and (iii) practice time in the prior month. We then fitted a series of linear regression models to test how well active practice experience predicted each outcome measure. Then, to test for a possible non-linear relationship between accumulated lifetime practice hours and outcome measures, we fitted equivalent generalized additive models (GAMs) and compared model fit to linear models with AIC and *R*^2^ values. We also conducted a visual inspection of the plotted GAM models to assess non-linear shape. For linear regression and GAM models, each outcome was tested independently with relevant demographic and personal characteristics entered as covariates. Stepwise regression analyses were then performed to identify the aspects of practice that most strongly predicted each outcome variable. Variables were processed using a backwards sequential approach with the stepAIC function from the MAAS R package (Venables & Ripley, 2002). Variables were binary unless otherwise specified and included demographic characteristics, age (continuous), gender, self-reported mental health diagnosis, recent experience of a major stressful life event, practice type, faith/tradition practiced within, tools and methods employed to support practice, retreat experience, and whether the participant meditated at a regular time each day (“regular time”) (for details, see Supplementary Table S1). A further analysis was conducted on those participants that reported using a mobile application, to assess whether the use of a particular app was associated with the relevant outcome variable.

For all stepwise regression models, zero-order correlations were reported for all statistically significant predictors. In all models, non-binary variables were standardized using the “scale” function from R.

All data analyses were completed using R version 3.6.1 in RStudio version 1.2.5001). Significance was set at *p* < 0.05. Nominal alpha was set at *p* < 0.05, and adjusted for multiple comparisons, where appropriate. No adjustment was made to correlations. Multivariable linear regression (with and without the smoothing variable) was implemented on five outcome measures, with adjustment at the model level for multiple comparisons using false discovery rate (Benjamini & Hochberg, 1995). As stepwise regressions were exploratory in nature, no correction was made.

## Results

### Participant Demographic and Practice Characteristics

**Fig. 1:**
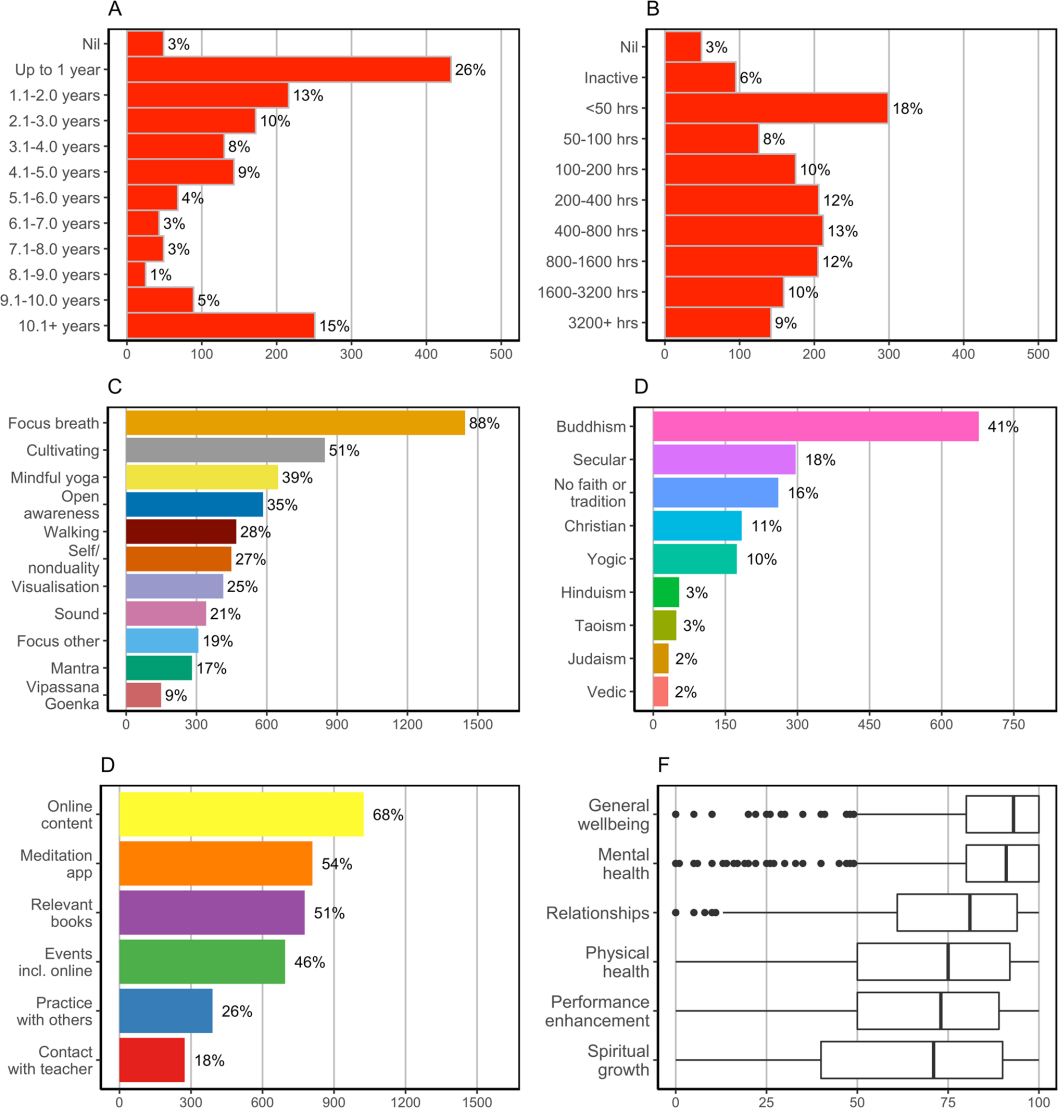
Summary of practice experience and characteristics. Panels **A** and **B** report the distribution of participants in terms of historical practice by duration of active practice (months/years) (**A**) and accumulated practice hours (**B**). Panel **C** reports practice types reported to be used by participants. Panel **D** reports faiths or traditions (if any) participants reported practice within. Panel **E** reports tools and methods participants reported using to support practice. Panel **F** reports the strength of different practice goals on a 100-point scale of importance, where 100 = extremely important, 50 = moderately important, and 0 = of no importance. *Notes.* For panel **D**, “No faith or tradition” refers to participants who did not select any faith or tradition (including secular)

Figure 1 depicts the distribution of participants by historical practice experience in terms of (A) duration of active practice and (B) accumulated lifetime practice hours; (C) the proportion of participants reporting each practice type; (D) the faith or tradition within which participants practice; (E) the tools and methods used to support practice; and (F) the relative strength of different practice goals. Participants mostly practiced within a Buddhist (41%) or secular/non-religious (33%) context, with popular practice types including focused attention on the breath (practiced by 88% of participants), cultivating practices (51%), mindful yoga (39%), and open awareness (35%). Among the participants who provided a subjective rating to all listed practice goals (*n* = 1258), the most important goals (subjectively rated on a 100-point scale) were general wellbeing (*M* = 88.3, *SD* = 17.1) and mental health (*M* = 87.1, *SD* = 18.4), followed by improving relationships (*M* = 76.2, *SD* = 25.5), physical health (i.e. better sleep, *M* = 71.4, *SD* = 27.0), performance (i.e. cognitive) enhancement (*M* = 68.8, *SD* = 27.4), and spiritual growth (*M* = 64.5, *SD* = 32.8). The importance of spiritual growth tended to increase for participants with more historical practice experience. For participants with at least 5 years of active practice (*n* = 475) for instance, the mean subjective rating of spiritual growth increased to 73.5 (*SD* = 29.0). Additionally, the correlation coefficient between accumulated practice hours and the subjective rating of spiritual growth was *r* = 0.25 while for all other goals, the correlation was either negative (mental health *r* =  − 0.19, performance enhancement *r* =  − 0.13, general wellbeing *r* = 0.06) or not statistically significant.

Table 2 reports psychological and personality characteristics of the participants and compares those results with comparative samples. Our sample was within 0.3 standard deviations of other representative samples for measures of affect (*n* = 1655; (Diener et al., 2009; Howell & Buro, 2015; Rahm et al., 2017) and psychological distress (*n* = 4527; (French et al., 2020; Klein et al., 2020; Ryan et al., 2010). For satisfaction with life, our sample was 0.89 standard deviations below the comparative sample (Cheung & Lucas, 2014). For measures of personality traits, our sample was ≤ 0.3 standard deviations of the weighted averages from the combined samples from Soto and John (2017b, *n* = 1459) for conscientiousness, extraversion, and negative emotionality. For open-mindedness and agreeableness, our sample was 0.48 and 0.59 standard deviations higher than the comparative sample (Soto & John, 2017b).

**Table 2:** Outcome variable scores for this study and comparative samples

Measure	Score range	Missing	Our study		Comparative samples	
			*n*	Mean (*SD*)	*n*	Mean (*SD*)
Psychological distress	10 to 50	115	1553	19.12 (6.57)	4527	21.12 (8.39)
Positive affect	6 to 30	108	1560	21.16 (4.41)	1655	22.23 (3.67)
Negative affect	6 to 30	101	1567	15.47 (4.33)	1665	15.17 (4.07)
Affect balance	− 24 to 24	115	1553	5.69 (7.87)	1665	7.07 (7.08)
Satisfaction with life	1 to 4	3	1665	2.83 (0.82)	2261	3.39 (0.63)
Open-mindedness	1 to 6	261	1407	4.15 (0.68)	1459	3.84 (0.65)
Conscientiousness	1 to 6	253	1415	3.61 (0.87)	1459	3.43 (0.73)
Extraversion	1 to 6	275	1393	3.19 (0.87)	1459	3.24 (0.77)
Agreeableness	1 to 6	285	1383	4.04 (0.70)	1459	3.67 (0.63)
Neg. emotionality	1 to 6	272	1396	2.76 (1.05)	1459	3.01 (0.94)

Missing responses are the aggregate of participants that skipped the whole section or missed too many responses in the relevant section (i.e. 1 or more for SPANE or K10, 2 or more for each BFI domain)

Comparative studies include for psychological distress — French et al., 2020; Klein et al., 2020; Ryan et al., 2010. For personality, Soto & John, 2017b. For measures of affect — Diener et al., 2009; Howell & Buro, 2015; Rahm et al., 2017. For satisfaction with life — Cheung & Lucas, 2014

### Relationship Between Historical Practice and Psychological Outcomes

#### Correlations

As shown in Table 3, both measures of historical practice (duration of active practice in months/years and accumulated lifetime practice hours) were significantly correlated with all outcome variables. Correlations with prior practice ranged from *r* = 0.22 to *r* = 0.24 for life satisfaction, *r* = 0.20 to *r* = 0.25 for measures of affect, and *r* =  − 0.28 to *r* =  − 0.30 for psychological distress, and from *r* = 0.45 to *r* = 0.71 between outcome variables. Recent practice (hours in the prior month) was also correlated with outcome variables but to a smaller degree, ranging from *r* = 0.22 for positive affect to *r* = 0.26 for affect balance.

**Table 3:** Means, standard deviations, and zero-order correlations with confidence intervals

	*M*	*SD*	1	2	3	4	5	6	7
1. Practice hours in past month	9.88	10.45							
2. Duration of active practice (years)	5.25	5.78	.34** [.29, .38]						
3. Accumulated lifetime practice hours	855.9	1277	.65** [.63, .68]	.81** [.79, .83]					
4. Psychological distress	19.12	6.57	− .27** [− .32, − .23]	− .28** [− .33, − .23]	− .30** [− .34, − .25]				
5. Positive affect	21.16	4.41	.22** [.18, .27]	.25** [.21, .30]	.26** [.21, .31]	− .63** [− .66, − .60]			
6. Negative affect	15.47	4.33	− .24** [− .28, − .19]	− .18** [− .23, − .14]	− .24** [− .28, − .19]	.71** [.68, .73]	− .62** [− .65, − .59]		
7. Affect balance	5.69	7.87	.26** [.21, .30]	.24** [.20, .29]	.28** [.23, .32]	− .74** [− .76, − .72]	.90** [.89, .91]	− .90** [− .91, − .89]	
8. Satisfaction with life	2.83	0.82	.23** [.19, .28]	.22** [.18, .27]	.25** [.20, .25]	− .50** [− .53,. − 46]	.56** [.52, .59]	− .45** [− .49, − .41]	.56** [.52, .59]

*M* and *SD* are used to represent mean and standard deviation, respectively

The confidence interval is a plausible range of population correlations that could have caused the sample correlation (Cumming, 2014)

^*^ indicates *p* < .05. ** indicates *p* < .01

#### Linear Models

Outcome variables were significantly different by age (continuous) and gender (categorical, Supplementary Material S2); hence, we included age and gender as covariates in subsequent models, except for positive affect, for which we covaried by age only. For the main analysis, we fitted univariable linear regression models for each outcome variable, with accumulated practice hours as the predictor (with relevant covariate/s). We excluded 94 participants from analysis when accumulated practice hours was used as the predictor because they did not meditate in the month prior to completing the survey (meditation time in the prior month was needed to calculate lifetime accumulated practice hours). For all outcome variables, accumulated practice hours predicted higher life satisfaction, positive affect, and affect balance, and lower psychological distress and negative affect. All results remained significant after correcting for multiple comparisons using FDR. Standardized beta coefficients ranged in magnitude from *β* = 0.177 for negative affect to *β* = 0.269 for positive affect. As results indicated no material difference between raw and log-transformed data for psychological distress, raw data are hereinafter reported for ease in interpretation. Regression summaries are reported in Table 4.

**Table 4:** Multivariable linear regression results with accumulated practice hours predicting outcome measures

Variable/predictors	*β*	95% C.I	*t*	*p*	*R*^2^
Psychological distress					
Age	− 0.189***	[− 0.138 to − 0.238]	− 7.44	< .001	.127
Gender	− 0.253***	[− 0.148 to − 0.36]	− 4.69	< .001	
Accumulated practice	− 0.218***	[− 0.169 to 0.269]	− 8.52	< .001	
Positive affect					
Age	− 0.028	[− 0.079 to 0.022]	− 1.11	.267	.069
Accumulated practice	− 0.270***	[0.219 to 0.32]	10.50	< .001	
Negative affect					
Age	− 0.096***	[− 0.045 to − 0.147]	− 3.69	< .001	.085
Gender	− 0.358***	[− 0.251 to − 0.467]	− 6.50	< .001	
Accumulated practice	− 0.177***	[− 0.126 to 0.229]	− 6.77	< .001	
Affect balance					
Age	0.036	[− 0.014 to 0.088]	1.39	.164	.085
Gender	0.188***	[0.081 to 0.298]	3.40	< .001	
Accumulated practice	0.253***	[0.201 to 0.305]	9.53	< .001	
Satisfaction with life					
Age	− 0.017	[− 0.065 to 0.035]	− 0.66	.506	.064
Gender	0.092	[− 0.015 to 0.195]	1.71	.087	
Accumulated practice	0.247	[0.195 to 0.295]	9.62	< .001	

*β*, standardized canonical coefficient; *95% C.I.*, 95% confidence interval; *t*, *t*-test value; *p*, *p*-value; *R*^*2*^, proportion of outcome variables variance explained by predictors

^*^ indicates *p* < .05. ** indicates *p* < .01. *** indicates *p* < .001

We additionally tested the strength of the relationship of each outcome variable with both accumulated practice hours and recent practice hours to gauge how these two measures of historical practice (i.e. one focusing on long-term historical practice and the other, recent practice) interact. These results are described in the Supplementary Materials (see S3 and Figure S1). For all outcome variables except negative affect, the interaction between recent practice time and accumulated lifetime practice was statistically significant. Moreover, main effects of both accumulated practice hours and recent practice hours were more pronounced in the interaction models than the simple models, indicating the importance of considering the interaction. The results indicate that recent practice has a greater effect on outcomes for those with less historical practice.

#### GAMs

**Fig. 2:**
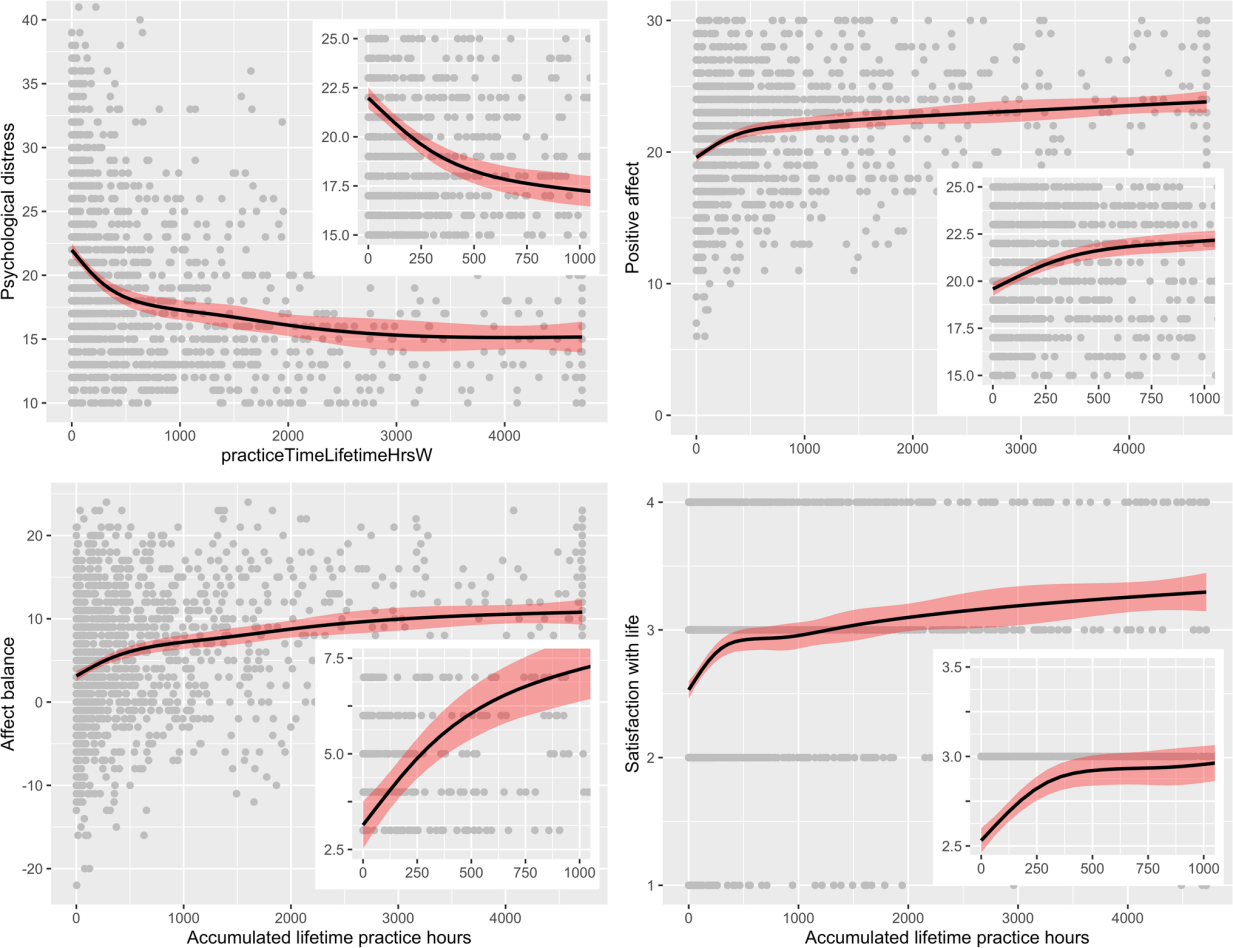
Visual representation of GAMs for psychological distress, positive affect, affect balance, and life satisfaction

We also fitted GAMs for each outcome measure (using the same covariates as described above) to test for the presence of a non-linear relationship, with smoothing only applied to the accumulated lifetime practice hours variable. To ascertain whether the smoothing of accumulated practice hours resulted in improved model fit, we compared equivalent GAMs with the only difference being the removal of the smoothing. Model fit improved in all cases, as indicated by reduced AIC, increased *R*^2^, and significance test using the “anova” function from R. These results are reported in Table 5. The improved model fit for each GAM suggests a non-linear relationship between accumulated practice time and outcome variables. The weakest evidence for a non-linear relationship is for negative affect, which failed a significance test after adjusting for multiple comparisons using FDR. The presence of a non-linear relationship is supported by the shape of the regression line in Fig. 2. A visual examination of these plots indicated that the slope of the curve is steepest in the early stages of practice, up to approximately 500 accumulated hours, before plateauing out to a shallower slope thereafter (although the size of error band increases at higher amounts of accumulated practice hours).

**Table 5:** Comparison of linear models and GAMs with smoothed term

Outcome measure	Smoothed GAM term	AIC	Deviance explained
Psychological distress	No	4194.758	.127
	Yes***	4151.062	.156
Positive affect	No	4311.377	.069
	Yes***	4271.487	.099
Negative affect	No	4306.063	.085
	Yes*	4299.986	.091
Affect balance	No	4267.300	.085
	Yes***	4245.202	.103
Life satisfaction	No	4612.269	.064
	Yes***	4574.056	.092

Smoothing occurs only on accumulated practice hours term in applicable models. Deviance explained in GAMs with no smoothed term is equal to *R*^2^ values in Table 4

^*^ indicates *p* < .05. ** indicates *p* < .01. *** indicates *p* < .001

To contextualize these results, we examined accumulated practice hours needed to achieve a change in measured outcomes that could be considered clinically relevant (based on a “minimal important difference” score from Cuijpers et al. (2014), estimated to be a standardized mean difference (SMD) of 0.24). In conducting this analysis, we restricted our sample to participants with less than 5 years of active practice (68.6% of the sample) to account for the diminishing returns that appear relevant for longer-term practice past a certain time threshold, as per Fig. 2. We also unstandardized the accumulated practice hours variable and multiplied it by 1000 to enable the interpretation of resulting beta coefficients as temporal rates of change. From these results, summarized in Table 6, we estimate it may take approximately 424 h of practice to achieve a “minimal important difference” (i.e. SMD of 0.24) for psychological distress, 497 h for satisfaction with life, 606 h for positive affect, 609 h for affect balance, and 811 h for negative affect.

**Table 6:** Estimated hours of practice to achieve 0.24 standardized mean difference improvement

Outcome measure	*n*	*β* (95% C.I.)	Hours to 0.24 SMD (95% C.I.)
Psychological distress	951	− 0.568 (− 0.400 to − 0.737)	422.5 (32,565 to 600.0)
Positive affect	958	0.415 (0.246 to 0.583)	578.3 (411.7 to 975.6)
Negative affect	960	− 0.298 (− 0.133 to − 0.463)	805.4 (518.4 to 1804.5)
Affect balance	956	0.396 (0.230 to 0.563)	606.1 (426.3 to 1043.5)
Life satisfaction	1021	0.485 (0.323 to 0.647)	494.8 (370.9 to 743.0)

*SMD*, standardized mean difference. 0.24 SMD threshold is from Cuijpers et al. (2014). *β*, standardized canonical coefficient, although accumulated practice hours predictor is unstandardized and multiplied by 1000 to allow for interpretation of *β* as temporal rates of change

### Exploring the Strongest Predictors of Practice on Favorable Outcomes

We then used multivariable stepwise multiple regression analyses to test the predictive strength of various reported practice characteristics for each outcome measure, after controlling for relevant covariates (as per above models) as well as accumulated practice hours. Aspects of practice included in this analysis were practice type (choice of 14 common practice types), practice tradition (choice of 10 faiths/traditions), tools and methods used to help practice (choice of six), prior experience of a multi-day silent retreat (binary), and whether meditation is usually practiced at a regular time (binary). A rationale for these choices is provided in the Supplementary Materials (see S4, questions 8, 11, 14, 21, 23). For each outcome measure, adding these aspects of practice improved model fit, with the final models accounting for 16.4% of the variance for psychological distress, 10.5% of the variance for positive affect, 12.0% of the variance for negative affect, 11.6% of the variance for affect balance, and 9.0% of the variance for life satisfaction. These results are summarized in Table 7. Across all five outcome measures, Vipassana (taught by S.N. Goenka) was a significant predictor with standardized regression coefficients ranging in magnitude from *β* = 0.246 to *β* = 0.294. Two other practice types, mindful yoga and cultivating practices (compassion-focussed, loving kindness), were significant predictors of multiple outcome measures — practicing yoga predicted lower psychological distress and higher positive affect and affect balance, while cultivating practices predicted higher positive affect and life satisfaction. Aside from practice types, meditating at a regular time was a significant predictor of favorable outcomes for psychological distress, negative affect, and affect balance. Notably, for all measures except practicing at a regular time, the magnitude of standardized regression coefficients was materially different than zero-order correlations.

**Table 7:** Regression models results to identify aspects of practice predicting outcomes

Measure/predictor	***β***	95% C.I	*t*	*p*	*r*	*R*^2^
Psychological distress						
Practice — Vipassana (Goenka)	− 0.294	(− 0.467 to − 0.122)	− 3.35	< .001	− .138	.164
Tools and methods — online	− 0.240	(− 0.373 to − 0.106)	− 3.46	< .001	− .096	
Tools and methods — events *	0.179	(0.050 to 0.308)	2.71	.007	− .040	
Practice at regular time	− 0.174	(− 0.273 to − 0.075)	− 3.52	< .001	− .171	
Retreat experience	− 0.126	(− 0.241 to − 0.012)	− 1.98	.047	− .219	
Practice — walking*	0.118	(0.010 to 0.226)	2.05	.040	− .005	
Practice — yoga	− 0.116	(− 0.219 to − 0.013)	3.22	< .027	− .011	
Positive affect						
Practice — Vipassana (Goenka)	0.246	(0.076 to 0.416)	2.84	.005	.115	.105
Tradition — Yogic	0.177	(0.009 to 0.346)	2.06	.039	.104	
Practice — focus other**	0.161	(0.029 to 0.293)	2.40	.017	.092	
Practice — sound	0.136	(0.010 to 0.262)	2.12	.034	.079	
Practice — yoga	0.125	(0.012 to 0.238)	2.17	.030	.091	
Practice — cultivating	0.117	(0.011 to 0.223)	2.17	.030	.136	
Negative affect						
Practice — Vipassana (Goenka)	− 0.265	(− 0.435 to − 0.094)	− 3.04	.002	− .128	.120
Practice at regular time	− 0.141	(− 0.243 to − 0.039)	− 2.72	.007	− .135	
Tools and methods — app*	0.141	(0.039 to 0.243)	2.71	.007	.120	
Tools and methods — events *	0.121	(0.015 to 0.226)	2.25	.025	.035	
Tradition — Buddhism*	0.113	(0.003 to 0.224)	2.02	.044	− .049	
Affect balance						
Practice — Vipassana (Goenka)	0.266	(0.094 to 0.438)	3.03	.002	.135	.116
Tools and methods — events*	− 0.155	(− 0.286 to − 0.025)	− 2.33	.020	.009	
Practice at regular time	0.148	(0.046 to 0.249)	2.84	.005	.147	
Tools and methods — online	0.140	(0.002 to 0.277)	2.00	.046	.049	
Practice — yoga	0.122	(0.007 to 0.237)	2.07	.038	.046	
Satisfaction with life						
Practice — Vipassana (Goenka)	0.250	(0.082 to 0.418)	2.92	.004	.127	.092
Practice at regular time	0.150	(0.049 to 0.250)	2.92	.004	.132	
Tools and methods — online	0.149	(0.013 to 0.285)	2.15	.031	.070	
Practice — cultivating	0.144	(0.042 to 0.246)	2.77	.006	.144	
Practice — yoga	0.125	(0.021 to 0.228)	2.37	.018	.048	
Practice — self enquiry	0.110	(0.001 to 0.219)	1.98	.048	.090	

*β*, standardized canonical coefficient; *95% C.I.*, 95% confidence interval; *t*, *t*-test value; *r*, zero-order correlations; *R*^*2*^, proportion of outcome variables variance explained by predictors

^*^Directionality of *β* and *r* suggests association with worse outcomes

^**^ “Focus other” refers to meditation with a focus on something other than the breath (e.g. a question, candlelight, rock)

While the use of a meditation app use was not a significant predictor of favorable psychological outcomes, we tested whether the use of any particular mobile application predicted favorable outcomes among the subset of participants that reported using an app within the past month. Table S4 shows a frequency distribution table of apps reported by more than *n* = 10 participants. This showed that the use of the *Waking Up* app (*n* = 109, 12.1% of users) was a significant predictor of favorable outcomes for psychological distress (*β* =  − 0.227, *p* = 0.043), negative affect (*β* =  − 0.288, *p* = 0.009), affect balance (*β* = 0.272, *p* = 0.015), and satisfaction with life (*β* = 0.215, *p* = 0.039). Additionally, the use of the *Plum Village* app (*n* = 36, 4.0% of users) was a significant predictor of higher life satisfaction (*β* = 0.380, *p* = 0.029), though we have less confidence in this result due to the low number of users.

## Discussion

This study had three principal aims. Aim 1 was to report the practice characteristics of a broad cross-section of contemporary meditation practitioners. Our sample featured a mix of naïve and experienced meditation practitioners predominantly practicing within a Buddhist (41%) or secular/non-religious (34%) context. Several practice types were widely reported including focussed attention on the breath (87%), cultivating practices (51%), yoga (39%), and open awareness (35%). Of the various tools and methods used to support practice, the most common were engaging with online content (68%) and using a meditation app (54%). The practice goals of highest relative importance were general wellbeing and mental health. Such characteristics are broadly consistent with the aims, goals, and recommendations of the modern mindfulness movement (Bodhi, 2016), which has advanced in successive stages beginning with pioneers within the insight tradition, continuing with the establishment of programs like MBSR (which features all of the abovementioned practices) and more recently, via the multitude of (largely commercial) online programs, and practices (McMahan & Braun, 2017). Given that much of the empirical research has been conducted in the context of MBPs, our findings suggest that the suite of practices that are used in such programs is broadly representative of how many contemporary meditators practice.

Aim 2 was to examine the relationship between measures of psychological outcomes and historical meditation practice experience. Our findings suggest that historical practice (measured by accumulated lifetime hours, after controlling for relevant demographic characteristics) was associated with favorable psychological outcomes. The strength of that association ranged from *β* = 0.269 for positive affect to *β* = 0.177 for negative affect. Prior studies have found associations of a similar magnitude between meditation experience (measured in years) and psychological wellbeing (*r* = 0.20) (Baer et al., 2012). A further analysis suggested the relationship between accumulated practice hours and favorable psychological outcomes may be non-linear, with the relationship being strongest for approximately the first 500 h of practice before plateauing. And in contrast to findings from randomized trials wherein clinically relevant changes are observed with approximately 40 h of structured practice (Khoury et al., 2013), we estimate that on average, it may take from approximately 400 to 800 h of self-directed practice to achieve a clinically relevant amount of change. Thus, it is possible that the considerable heterogeneity in how meditation is practiced outside of standardized programs and trials may reduce the magnitude of effects and/or lengthen the amount of practice required to observe changes. Although the magnitude of change from self-directed practice, on average, may not reach clinical relevance until approximately 400 h, smaller but still relevant changes are likely to occur with fewer practice hours, which may motivate people to continue practicing.

We also compared the strength of the relationship between historical meditation practice experience (measured as accumulated lifetime practice hours) and recent past practice hours, observing that historical meditation practice experience was a stronger predictor of outcome variables than recent practice for all outcomes except negative affect. Additionally, we observed a statistically significant interaction between historical practice experience and recent practice for those same outcome measures, also evident in the non-parallel lines depicted in Figure S1. Together, these findings suggest the relationship between recent practice and psychological outcomes may be mediated by experience/proficiency. In other words, recent practice may be relatively more important to psychological outcomes for inexperienced practitioners than for experienced practitioners, with experienced practitioners possibly benefiting from the greater amount of historical practice they have undertaken.

The remainder of this section describes findings from Aim 3, which was to report the practice characteristics that best predicted favorable psychological outcomes. Several practice types were associated with favorable outcomes across several domains, most prominently Goenka-style Vipassana (*n* = 146), yoga (*n* = 650), and cultivating practices (*n* = 750). The other main characteristic of practice to predict favorable outcomes was meditating at a regular time (*n* = 732). There is limited evidence in the broader literature for the benefits of practicing Goenka’s version of Vipassana, which is principally a body-scan technique (Anālayo, 2011) constituting one of the two main original lineages of Buddhist-influenced meditation practice to expand beyond Asia (Bodhi, 2016). In one study, Szekeres and Wertheim (2015) found significant improvements for wellbeing, stress, and self-reported mindfulness for people practicing this style of Vipassana, although this and similar research (Krygier et al., 2013) was in the context of an intensive retreat. More robust evidence is evident for the favorable effects of cultivating practices, which typically include compassion-based practices like self-compassion and loving-kindness (Neff & Pommier, 2013; Salzberg, 2002) that are typically orientated toward cultivating positive emotional states (Salzberg, 2002). While our study found an association between the use of cultivating practices for positive affect and life satisfaction, meta-analyses have found modest evidence of beneficial effects from practicing cultivating practices for positive emotions (Zeng et al., 2015) and stress, but not for life satisfaction, and negative emotions (Galante et al., 2014). And there is mixed evidence that cultivating practices are more effective at promoting positive emotions that other kinds of meditation. In two randomized controlled trials where the effects of loving kindness and compassion practices were tested against other forms of meditation (i.e. concentration and mindfulness), a significant between-group difference for positive emotions was evident in one study (May et al., 2014) but not the other (Koopmann-Holm et al., 2013).

The potential benefits of mindful yoga have perhaps been most rigorously studied in the context of MBPs, with the practice constituting one of several practices within a standard MBSR program (Kabat-Zinn, 2011). Although evidence is lacking for the beneficial effects of yoga independent to other aspects of these programs (Carmody & Baer, 2008), one study found that participants practicing yoga at home instead of seated meditation experienced a statistically significant reduction in anxiety, suggesting these practices may have similarly beneficial effects (Quach et al., 2017). While other research suggests yoga-based interventions may provide benefits for various aspects of psychological wellbeing (Frank et al., 2017), better quality studies are needed to confirm such effects (Domingues, 2018; Luu & Hall, 2017).

While the associations between reported use of Goenka-style Vipassana and cultivating practices provide some support for their efficacy, it is important to consider a high potential for survivorship bias. In other words, those individuals who most benefitted from the practice may represent those individuals who have continued to use the practice. Combined with the fact that Vipassana practitioners represented less than 10% of the sample (*n* = 146) and exhibited a mean practice amount 0.5 standard deviations higher than the overall mean, it is possible that we mostly captured those for whom the practice led to positive results. One distinctive feature of Vipassana practitioners relates to prior experience in a silent retreat — given that a 10-day retreat represents the introduction to this practice type for most people (Anālayo, 2011), 84.2% of participants practicing within this tradition reported prior experience attending a multi-day intensive retreat. By comparison, 36.0% of remaining participants reported prior participation in a multi-day retreat. Participants using cultivating practices also reported prior participation in a retreat at higher rates than other participants — 49.4% compared to 32.7%. The intensive nature of practice within meditation retreats therefore may provide greater benefits than what might be predicted by the time investment alone, for instance, by allowing for the development of a high degree of concentration, mindfulness, and similar meditative faculties (Goldstein & Kornfield, 2001). Intensive practice has, however, also been associated with a higher likelihood of experiencing negative or adverse effects from meditation practice (Lindahl et al., 2017; Schlosser et al., 2019), and therefore engaging in intensive practice may entail not only additional benefits but also risks.

Participants reporting the use of these practices also generally reported more accumulated practice hours than participants not using those practices; Vipassana practitioners had mean accumulated practice hours of 1986 (*SD* = 3392) compared to 1466 (*SD* = 2255) for other participants, while participants using cultivating practices had mean accumulated practice hours of 1421 (*SD* = 2713) compared to 810 h (*SD* = 1941) for other participants. People meditating at a regular time each day were also more experienced than those who did not, with 1558 h of practice over 7.4 years compared to 719 h over 4.6 years. For users of meditation-related mobile applications, the use of the *Waking Up* app was associated with beneficial outcomes in four of the five measured domains (psychological distress, negative affect, affect balance, satisfaction with life). *Waking Up* users represented 12.1% of app-using participants (*n* = 109) and were mostly male, representing 64.2% of *Waking Up* users compared to 30.1% across the sample (29% for app users). Across all participants, app users had approximately half the amount of accumulated practice hours (*M* = 722, *SD* = 1459) compared to participants that did not use an app (*M* = 1544, *SD* = 3065) — a characteristic that was also consistent with the profile of *Waking Up* users (*M* = 717, *SD* = 1975).

This study had two key strengths: namely including a large and broadly representative sample of contemporary meditation practitioners in a cross-sectional design, enabling us to explore the effects of meditation over a longer time horizon than MBPs and other short-term interventions, and at a higher level of granularity than prior cross-sectional work. Using this approach, we were able to detect evidence of non-linear change at different stages of meditative proficiency and estimate the time of self-directed practice that may be needed to achieve a clinically relevant amount of change. We believe these are valuable contributions to the dose–response literature for meditation.

### Limitations and Future Directions

The study has a few limitations worth noting. First, the retrospective cross-sectional design precludes our ability to make causal inferences about the impact of meditation on psychological outcomes. So, while meditation practice may lead to improved psychological outcomes, it is also possible that people with better mental health may be more likely to engage in meditation, and/or sustain a meditation practice over a relatively long-time duration. Relatedly, our results may also be subject to survivorship bias — given that a large proportion of our sample are active meditators, these may be people who have most benefitted from the practice and thus continued to practice (in some cases) for a sustained period. To confirm these effects, a prospective longitudinal study design that incorporates an intent-to-treat analysis is necessary that follows people’s practice over an extended duration. Since research evaluations of MBPs typically feature less than 50 h of total practice time, little is known about the benefits that may accrue over longer time durations. However, there are inherent experimental and logistical difficulties associated with studying the effects of longer-term meditation in controlled experimental conditions, particularly when starting with relative novice practitioners who are randomized to a condition that requires them to maintain a regular practice over extended periods. One way to manage this may be to measure the effects of meditation in a prospective longitudinal design in people who already have an established meditation practice to measure whether people continue to practice and if so, whether the effects of meditation continue accruing in the monitored period in people who already have a substantial amount of accumulated practice time.

The second limitation is that although our sample is international, it is by no means representative of meditators globally, being heavily weighted toward high-income English-speaking countries, with 93% of participants residing in Australia, the USA, the UK, the European Union, and Canada. Although the remaining participants come from a diverse array of countries (e.g. 3.2% are from Singapore, India, China, Brazil, the remaining 3.8% are from 24 different countries), their overall small proportion means our results cannot be generalized to meditators globally. We do, however, have some confidence that our sample is broadly representative of engaged meditation practitioners within the countries where our recruitment was targeted, based on our large sample size and the fact that our targeted sample and convenience sample are broadly similar across several key variables (see Supplementary Table S1). Future research of a similar nature in meditators that are not well represented in our sample (i.e. non-English speaking, non-Western) is necessary to confirm the generalizability of our findings.

The third limitation relates to our estimate of accumulated practice time, which is based on an extrapolation of the participants’ self-reported duration and frequency of practice within the prior month. Direct indicators of prior practice would be more reliable, which may be increasingly possible in the future as more people use fitness tracking technologies to help maintain a record of practice. Although we tried to collect such data from participants who use the Insight Timer app, we did not receive enough data to warrant inclusion.

The fourth limitation is that the study’s outcome variables are not extensive and do not perfectly reflect the relative importance of different practice goals of our participants, or of meditators generally. The potential incongruence between goals and measures may be particularly relevant for practitioners with more experience who — as our and others’ Sedlmeier and Theumer (2020) data show — tend to place a higher value on spirituality-orientated practice goals. In contrast, we focussed our outcomes on commonly used indicators of affect, wellbeing, and mental health (Diener et al., 2009; Kessler et al., 2002). It is important to note therefore that such a focus might create a bias toward those who practice for mental health-related reasons and/or are earlier in their meditative development. In other words, those experiences most sensitive to change at different stages of meditation may vary considerably (Sedlmeier & Theumer, 2020). Therefore, while our data show a plateauing of benefits after approximately 500 h, this may instead reflect a shift in the orientation of practice toward aspects of psychological wellbeing that were not measured. In order to detect changes resulting from meditation in more experienced practitioners, future research may benefit from the use of measures targeting those qualities that tend to motivate the practice of such practitioners, including such traits as equanimity, decentring, and self-transcendence (Desbordes et al., 2014; Yaden et al., 2017).

The fifth limitation is the potential inconsistency across participants to accurately identify the type of meditation they practice, and the quality of the practice they engage in (Del Re et al., 2013). Sixth, as all data was self-reported, correlational findings could have been inflated by common method biases like response styles, social desirability, and priming effects (e.g. Podsakoff et al., 2003). And the final limitation we note is that we did not ask participants to specify whether and to what degree prior practice they had undertaken was individual self-directed practice, or in a group setting. The context of practice may affect how benefits accumulate over time and would be worth exploring in future research.

Thus overall, we found evidence of non-linear dose–response effects between historical meditation hours and psychological outcomes, with the strongest gains extending beyond the timeframe of meditation app programs, brief MBPs and standard MBPs, up to 500 h of practice. Practice types, including Vipassana (as taught by S.N. Goenka) and cultivating practices (e.g. compassion, lovingkindness), were more strongly predictive of favorable psychological outcomes, suggesting their potential utility for ongoing study. Importantly, except for *Waking Up*, meditation apps were not associated with significant improvements in psychological outcomes or sustained practice. These results make an important contribution to the literature on the dose–response effects of meditation by estimating the strength and nature of the association between practice time and outcomes that accrue over longer time durations than are represented in experimental work, and across a variety of practice modalities and doses.

## Supplementary Information

Below is the link to the electronic supplementary material.

Supplementary file1 (PDF 159 KB)

Supplementary file2 (DOCX 323 KB)

## Data Availability

Data are available at the Open Science Framework (https://osf.io/zbqdh/).
