# Supplementary material for: Dose–response Relationship of Reported Lifetime Meditation Practice with Mental Health and Wellbeing: a Cross-sectional Study
Source: Mindfulness (N Y). 2022 Sep 28;13(10):2529–46. doi: 10.1007/s12671-022-01977-6 (PMC9517970; doi:10.1007/s12671-022-01977-6)
Supplement: Supplementary file 1 — Supplementary file1 (PDF 159 KB) [file 12671_2022_1977_MOESM1_ESM.pdf]

Supplementary Information

S1. Comparison of key variables for targeted and convenience sample

Table S1 summarizes the characteristics of the targeted and convenience sample. Chi-squared results show significant differences for only age and retreat experience.

****Table S1 here****

S2. Analyses to determine variables to covary in main analysis

Correlations between age and all outcome measures were statistically significant, ranging from weak correlations of r=.05 for positive affect (p=.033) and r=.06 for satisfaction with life positive affect (p= to -.022) to stronger correlations for negative affect of r=-.13 (p<.001) and r=-.24 (p<.001) for psychological distress. Age was more strongly correlated with practice experience – r=.31 for accumulated practice hours and r=.42 for practice years, which reflects the greater opportunity people of older ages have had to practice meditation. Age was therefore included as a covariate for all subsequent models.

For gender, *t* tests showed significant differences between males and females for all outcome measures except positive affect, and therefore gender was included as a covariate in all subsequent models where a significant difference was evident.

For country of residence, reported countries were grouped into four categories to test for differences – Australia (n=844), North America (n=484), United Kingdom (n=140), and other (n=191). One-way ANOVA tests showed no significant differences across country groups for any of the measured variables.

S3. Analysis of interaction between accumulated lifetime practice and recent practice

****Insert Table S2 here****

**** Insert Figure S1 here ****

S4. Partial correlations

****Insert Table S3 here****

S5. Frequency distribution of reported mobile apps

****Insert Table S4 here****

S6. Survey Questions

Demographics

1. What is your preferred name?
2. What is your email address? (We need this to follow-up with you)
3. What is your age (in years)? *
4. What is your gender? [Y/N]
5. In which country (or union of countries) do you live (primary country of residence)?
6. Do you have any diagnosed mental health or neurological conditions? [Y/N]
7. Would you be OK with us contacting you in the future about related projects? [Y/N]

Meditation history

8. For how long have you been actively practicing meditation? [text box]

   [Try to estimate active time. For example, if you started 12 years ago, but didn't practice for 4 years, you would enter 8 years.] *

9. What types of contemplative practices do you use on a regular basis? [tick all that apply]

   - ☐ Cultivating (e.g., loving kindness [metta], (self-)compassion, Brahmaviharas)
   - ☐ Focused attention on the breath (e.g. Samatha)
   - ☐ Focused attention on something other than the breath (e.g., a question, candlelight, rock)
   - ☐ Gentle repetition - mantra (Transcendental meditation, Vedic)
   - ☐ Movement practice – pranayama
   - ☐ Movement practice - Qi Gong / Tai Chi
   - ☐ Movement practice - walking meditation (slow, deliberate walk)

- ☐ Movement practice - yoga (with considerable focus on the breathe, body sensations etc.)
- ☐ Open awareness (e.g., Insight / Vipassana)
- ☐ Self enquiry/observation (e.g., non-dual awareness, kōan)
- ☐ Sound meditation
- ☐ Visualisation (e.g., guided imagery)
- ☐ Vipassana (as taught by S.N. Goenka)

10. Do you practice within a particular faith/tradition? If so, tick the box(es) that apply below.
    - ☐ Buddhism
    - ☐ Christianity
    - ☐ Hinduism
    - ☐ Judaism
    - ☐ Islam
    - ☐ Secular
    - ☐ Shinto
    - ☐ Taoism
    - ☐ Vedic
    - ☐ Yogic
    - ☐ Other __________
11. How important are each of the below goals for you in your meditation practice? *[degree of importance indicated on slider from 1 to 100, 1='Not important', 50='Moderately important', 100='Extremely important']*
    - Spiritual growth (e.g. pursuing enlightenment)
    - Mental health (e.g. manage stress/anxiety)
    - Physical health (e.g. sleep better)
    - Performance enhancement (e.g. improved cognition, productivity)
    - Improve relationships (e.g. be a better spouse, friend, parent, etc.)

- General well-being (e.g. become more emotionally balanced and calm)

12. What is the minimum time duration you believe you need to meditate to achieve the goal/s of your practice? [select one option, and specify the time period if relevant]
    - A number of days (specify number) [text box]
    - A number of weeks (specify number) [text box]
    - A number of months (specify number) [text box]
    - Ongoing/lifetime
13. How much time do you believe you need to meditate each week to achieve the goal/s of your practice? [text box]
14. Have you ever participated in a multi-day, silent meditation retreat? [Y/N]
15. Do you use a meditation app to help you practice? [Y/N]
16. Which meditation app/s do you use?
    - ☐ 10% Happier
    - ☐ Calm
    - ☐ Headspace
    - ☐ Insight Timer
    - ☐ Openground
    - ☐ Smiling Mind
    - ☐ MyLife (Stop,Breathe & Think)
    - ☐ Plum Village
    - ☐ Waking Up
    - ☐ Other __________

Recent practice

17. Did you meditate* in the past month? [Y/N]

    * For this study, meditation constitutes any practice inspired by or consistent with the general principles of paying attention to your experience, on purpose, in the present moment, with acceptance/non-judgment/discernment)

18. In the past month, approximately how many days (on average) did you meditate each week? [1-7]
19. On the days that you meditated in the past month, how much time (on average) did you spend meditating?
    - 15 minutes or less
    - 16 to 30 minutes
    - 31 to 45 minutes
    - 46 to 60 minutes
    - More than 60 minutes
20. How does this amount of meditation compare to past periods when you have been actively meditating?

[time indicated in a slider from 1 to 100, 1 = 'A lot less', 50 = 'About the same', 100 = 'A lot more']

21. In the past month, what tools or methods did you use to support your meditation practice? (tick as many that apply)
    - ☐ Meditation app
    - ☐ Participation in events with other meditators (incl. life online events)
    - ☐ Reading spiritual or meditation-related books
    - ☐ Engagement with online content (i.e. blogs, videos, podcasts)
    - ☐ Personal communication with a meditation teacher or spiritual figure
    - ☐ Practicing meditation with others (i.e. friends)
22. In the past month, what percentage of your meditation sessions were guided (i.e. with audio instructions during practice)?

[% of guided sessions indicated in a slider from 0 to 100, 0 = 'All unguided', 100 = 'All guided']

23. In the past month, have you meditated at a regular time each day? [Y/N]

Stressful event

24. In the past month, have you experienced a major stressful life event? [Y/N]
25. Was this stressful event related to COVID-19? [Y/N]
26. Please indicate on a scale of 1 to 5 how stressful that event was.

[Likert scale, 1='A little stressful', 3='Moderately stressful', 5='Extremely stressful']

Option to continue

27. Thank you for the time you have taken so far. The next section of the survey is optional. It includes three sections, each of which includes different psychological measures. It should take about 2 minutes to complete. Do you wish to continue? [Y/N]
