# Supplementary material for: Dose–response Relationship of Reported Lifetime Meditation Practice with Mental Health and Wellbeing: a Cross-sectional Study
Source: Mindfulness (N Y). 2022 Sep 28;13(10):2529–46. doi: 10.1007/s12671-022-01977-6 (PMC9517970; doi:10.1007/s12671-022-01977-6)
Supplement: Supplementary file 2 — Supplementary file2 (DOCX 323 KB) [file 12671_2022_1977_MOESM2_ESM.docx]

**Figure S1** Interaction plots showing differential outcomes based on recent practice


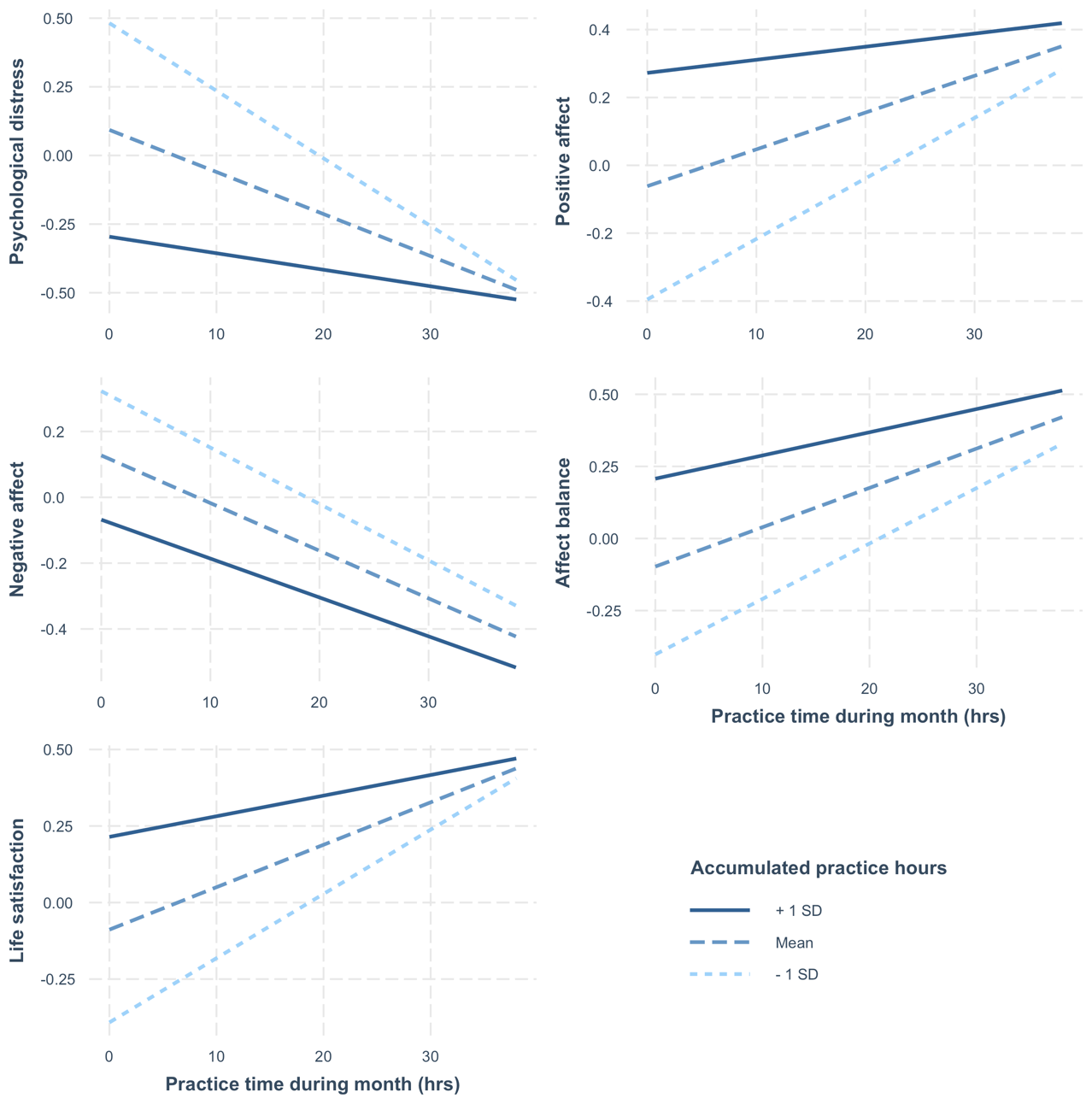


*Note.* Interaction effect is statistically significant for psychological distress (p<.001), positive affect (p<.001), affect balance (p=.011), and satisfaction with life (p<.001).

Y-axis is standardized for all outcome variables.

| **Table S1** Comparison of key variables for Facebook-recruited participants | | | |
| --- | --- | --- | --- |
| Variable | Targeted sample | Convenience sample | Test statistic |
|  | n (%) | n (%) |  |
| Total participants | 251 (15.1) | 1413 (84.9) |  |
| Gender – Female | n (70.5) | n (69.8) | χ^2^(1)=0.03 |
| Retreat experience | n (25.5) | n (42.9) | χ^2^(1)=27.71*** |
|  | *M* (*SD*) | *M* (*SD*) |  |
| Age | 49.8 (14.7) | 44.6 (15.2) | χ^2^(57)= 77.76* |
| Active practice years | 6.1 (9.3) | 5.8 (7.4) | χ^2^(164)=179.16 |
| Accumulated practice hours | 748.7 (1326.8) | 876.3 (1268.8) | χ^2^(1242)=1136.0 |
| Psychological distress (K10) | 19.54 (7.2) | 19.0 (6.5) | χ^2^(34)=35.60 |
| Positive affect (SPANE) | 20.42 (4.4) | 21.3 (4.4) | χ^2^(24)=28.31 |
| Negative affect (SPANE) | 15.1 (4.2) | 15.5 (4.4) | χ^2^(23)=18.88 |
| Affect balance (SPANE) | 5.3 (7.9) | 5.8 (7.9) | χ^2^(42)=29.10 |
| Satisfaction with life (SWLS) | 2.7 (0.8) | 2.9 (0.8) | χ^2^(3)=6.95 |
| *Note.* Targeted sample was recruited via Facebook in a one-month campaign.  * indicates p < .05. ** indicates p < .01. *** indicates p<.001 | | | |

| **Table S2** Multivariable linear regression results with accumulated lifetime and past month practice hours predicting outcome measures | | | | | | |
| --- | --- | --- | --- | --- | --- | --- |
| Measure / predictor | Model | **β** | 95% C.I. | *t* | *p* | *R^2^* |
| **Psychological distress** | |  |  |  |  |  |
| Accumulated hours | Main effect | -0.210 | [-0.272 to -0.148] | -6.64 | <.001 | .100 |
| Past month hours | Main effect | -0.135 | [-0.196 to -0.073] | -4.27 | <.001 |  |
| Accumulated hours | Interaction | -0.297 | [-0.370 to -0.224] | -7.99 | <.001 | .111 |
| Past month hours | Interaction | -0.160 | [-0.223 to -0.098] | -5.02 | <.001 |  |
| Accumulated & past month | Interaction | 0.097 | [0.054 to 0.141] | 4.38 | <.001 |  |
| **Positive affect** |  |  |  |  |  |  |
| Accumulated hours | Main effect | 0.199 | [0.136 to 0.262] | 6.19 | <.001 | .073 |
| Past month hours | Main effect | 0.095 | [0.032 to 0.157] | 2.95 | .003 |  |
| Accumulated hours | Interaction | 0.265 | [0.190 to 0.339] | 6.95 | <.001 | .080 |
| Past month hours | Interaction | 0.113 | [0.050 to 0.177] | 3.49 | <.001 |  |
| Accumulated & past month | Interaction | -0.073 | [-0.117 to -0.029] | -3.23 | <.001 |  |
| **Negative affect** |  |  |  |  |  |  |
| Accumulated hours | Main effect | -0.144 | [-0.207 to -0.081] | -4.49 | <.001 | .069 |
| Past month hours | Main effect | -0.144 | [-0.207 to -0.081] | -4.49 | <.001 |  |
| Accumulated hours | Interaction | -0.169 | [-0.243 to -0.095] | -4.46 | <.001 | .069 |
| Past month hours | Interaction | -0.151 | [-0.215 to -0.087] | -4.64 | <.001 |  |
| Accumulated & past month | Interaction | 0.028 | [-0.017 to 0.072] | 1.22 | .221 |  |
| **Affect balance** |  |  |  |  |  |  |
| Accumulated hours | Main effect | 0.197 | [0.134 to 0.260] | 6.12 | <.001 | .087 |
| Past month hours | Main effect | 0.128 | [0.066 to 0.191] | 4.02 | <.001 |  |
| Accumulated hours | Interaction | 0.250 | [0.175 to 0.324] | 6.55 | <.001 | .091 |
| Past month hours | Interaction | 0.142 | [0.079 to 0.206] | 4.41 | <.001 |  |
| Accumulated & past month | Interaction | -0.058 | [-0.103 to -0.014] | -2.57 | .010 |  |
| **Satisfaction with life** |  |  |  |  |  |  |
| Accumulated hours | Main effect | 0.166 | [0.105 to 0.227] | 5.31 | <.001 | .070 |
| Past month hours | Main effect | 0.126 | [0.064 to 0.187] | 4.02 | <.001 |  |
| Accumulated hours | Interaction | 0.233 | [0.160 to 0.305] | 6.32 | <.001 | .077 |
| Past month hours | Interaction | 0.145 | [0.083 to 0.207] | 4.57 | <.001 |  |
| Accumulated & past month | Interaction | -0.074 | [-0.117 to -0.032] | -3.41 | <.001 |  |
| *Note.* β **=** standardized canonical coefficient; 95 CI = 95% confidence interval; t = *t*-test value; p = p-value; R^2^ = proportion of outcome variables variance explained by predictors. | | | | | | |

| **Table S3** Means, standard deviations, and partial order correlations adjusting for age | | | | | | | | | |
| --- | --- | --- | --- | --- | --- | --- | --- | --- | --- |
|  | **M** | **SD** | **1** | **2** | **3** | **4** | **5** | **6** | **7** |
| 1. Practice hours past month | 9.88 | 10.45 |  |  |  |  |  |  |  |
| 2. Duration of active practice (years) | 5.25 | 5.78 | .31** |  |  |  |  |  |  |
| 3.Accumulated lifetime practice hours | 855.9 | 1277 | .65** | .78** |  |  |  |  |  |
| 4.Psychological distress | 19.12 | 6.57 | -.25** | -.21** | -.25** |  |  |  |  |
| 5. Positive affect | 21.16 | 4.41 | .22** | .26** | .26** | -.63** |  |  |  |
| 6. Negative affect | 15.47 | 4.33 | -.23** | -.15** | -.21** | .70** | -.62** |  |  |
| 7. Affect balance | 5.69 | 7.87 | .25** | .23** | .26** | -.74** | .90** | -.89** |  |
| 8. Satisfaction with life | 2.83 | 0.82 | .23** | .22** | .24** | -.50** | .56** | -.45** | .56** |
| *Note*. M and SD are used to represent mean and standard deviation, respectively.  * indicates p < .05. ** indicates p < .01. | | | | | | | | | |

| **Table S4** Frequency distribution of reported mobile apps | | |
| --- | --- | --- |
| **Mobile app** | **n** | **% of app users** |
| Insight Timer | 412 | 45.6 |
| Headspace | 185 | 20.5 |
| Calm | 138 | 15.3 |
| Waking Up | 109 | 12.1 |
| Smiling Mind | 93 | 10.3 |
| Ten Percent Happier | 61 | 6.8 |
| Openground | 37 | 4.1 |
| Plum Village | 36 | 4.0 |
| Dhamma.org | 15 | 1.7 |
| MyLife | 13 | 1.4 |
| *Note.* Only mobile apps reported by at least 10 participants are included. | | |
